# Upconversion Nanoparticles for Bioimaging and Regenerative Medicine

**DOI:** 10.3389/fbioe.2016.00047

**Published:** 2016-06-13

**Authors:** María González-Béjar, Laura Francés-Soriano, Julia Pérez-Prieto

**Affiliations:** ^1^Departamento de Química Orgánica, Instituto de Ciencia Molecular (ICMol), Universidad de Valencia, Valencia, Spain

**Keywords:** transparency, NIR excitation, upconverted (UV–VIS–NIR) emission, non-toxic nanoparticles, multi-wavelength/multimodal bioimaging, cell behavior regulation

## Abstract

Nanomaterials are proving useful for regenerative medicine in combination with stem cell therapy. Nanoparticles (NPs) can be administrated and targeted to desired tissues or organs and subsequently be used in non-invasive real-time visualization and tracking of cells by means of different imaging techniques, can act as therapeutic agent nanocarriers, and can also serve as scaffolds to guide the growth of new tissue. NPs can be of different chemical nature, such as gold, iron oxide, cadmium selenide, and carbon, and have the potential to be used in regenerative medicine. However, there are still many issues to be solved, such as toxicity, stability, and resident time. Upconversion NPs have relevant properties such as (i) low toxicity, (ii) capability to absorb light in an optical region where absorption in tissues is minimal and penetration is optimal (note they can also be designed to emit in the near-infrared region), and (iii) they can be used in multiplexing and multimodal imaging. An overview on the potentiality of upconversion materials in regenerative medicine is given.

## Introduction

Regenerative medicine aims to maintain, regenerate, and replace damaged or non-functional human cells, tissues, or organs to restore normal functions (Engel et al., 2008; Harrison and Sirivisoot, 2011; Gao et al., 2015; Mitragotri et al., 2015) *via* stimulating the body’s own repair mechanisms. In this context, nanostructures can play an important role regarding implants or scaffolds for tissue engineering and cell therapies, e.g., nanopatterning of surfaces to elicit specific biological responses from the host tissue and organs (Engel et al., 2008; Zhang and Webster, 2009; Harrison and Sirivisoot, 2011). Thus, the generation of new surfaces, structures, and materials containing nanoparticles (NPs) can provide the possibility of mimicking the natural environment of cells and promoting certain functions, such as cell mobility, cell adhesion, and cell differentiation that would be directly related to the nanotopography of the biomaterial (Engel et al., 2008).

The capability of nanomaterials to be multifunctional, as they can consist of different functional components in a single unit, is leading to significant advances over traditional imaging, sensing, and structural technologies (Harrison and Sirivisoot, 2011). Thus, NPs are used in biomedical applications for imaging (cell tracking and visualization), therapy, drug delivery aimed at target biological functions, surface modifications of implantable materials, diagnosis (Engel et al., 2008; Harrison and Sirivisoot, 2011; Gao et al., 2015), and even in the regulation of cell behavior (adhesion, growth, and differentiation), which is of relevance in regenerative medicine (Mitragotri et al., 2015).

New smart biomaterials could be implanted to monitor and direct the regenerative process at the cellular level. For example, NPs could help to monitor a disease (e.g., *via* emission or magnetism) and bring about tissue repair (e.g., through light-induced targeted delivery) creating better non-invasive regenerative therapies (Harrison and Sirivisoot, 2011; Gao et al., 2015). Interestingly, stem cells have the ability to generate all types of tissues together with an unlimited self-renewal capacity; hence, research is focused on being able to locate, recruit, and mark these cells to monitor and/or trigger the regeneration process (Harrison and Sirivisoot, 2011).

Nanoparticles are also being explored as nanocarriers for theranostic applications (Grazú et al., 2012; Muthu et al., 2014). This new field integrates NP design with simultaneous imaging and therapy, aiming to offer individualized treatments based on *in vivo* molecular images to allow for a comprehensive diagnosis (Rai et al., 2010). Several NPs have been used as platforms for NP-based theranostics (Choi et al., 2012; Miao et al., 2016): gold nanoparticles (GNPs) (Gao and Li, 2016), carbon nanotubes (CNTs) (Tran et al., 2009; Yun et al., 2012; Fraczek-Szczypta, 2014), magnetic NPs (MNPs) (Gao et al., 2015), silica NPs (SNPs) (Santra et al., 2005; Vivero-Escoto et al., 2012), quantum dots (QDs) (Ho and Leong, 2010), and upconversion NPs (UCNPs) (Chen et al., 2014a), among others. Remarkably, it has been estimated that at least half of the drugs used in 2020 will be based on nanotechnology (Grazú et al., 2012).

Current methods of evaluating cell treatments typically involve destructive or invasive techniques, such as tissue biopsies, whereas traditional non-invasive methods, such as magnetic resonance imaging (MRI) and positron emission tomography (PET), rely heavily on contrast agents and usually lack the specificity or resident time to be a viable option for cell tracking (Engel et al., 2008; Harrison and Sirivisoot, 2011). Photonic applications for diagnostics, therapy, and interventional guidance are increasing (Jin et al., 2011; Rwei et al., 2015). Fiber-optic based catheters can be used to perform localized imaging or laser ablation of a desired target to treat, for example, coronary artery disease (van Soest et al., 2015). Moreover, irradiation density is easy to dose and can provide spatiotemporal control (Rwei et al., 2015; van Soest et al., 2015). The limitations of biophotonic technologies for imaging usually arise from limited penetration depth of light into tissues; however, penetration depth could reach the centimeter scale for applications that rely on near-infrared (NIR) wavelengths and on optical power (diffuse optics and sensing) (Rwei et al., 2015; van Soest et al., 2015). UCNPs are transparent to visible light but can absorb two or more photons in the NIR region and emit at a higher energy level *via* a non-linear conversion process. Therefore, these luminescent NPs enable high-contrast optical biomedical imaging by suppressing the background of tissue autofluorescence and avoiding high absorption of the tissue.

In this mini-review, an overview of the use of NPs in regenerative medicine and what UCNPs can offer to this field is given.

## Nanoparticles in Biomedicine

Nanoparticles used in biomedicine must be biocompatible, water-dispersible, and stable in physiological media (Mu et al., 2014). To date, most of the ongoing nanomedicine clinical trials or those already on the market are being injected and provide passive drug targeting (Li et al., 2010; Grazú et al., 2012; Markovsky et al., 2012; Rwei et al., 2015). Therefore, the stability of nanocarriers in biological media is crucial when formulating nanomedicines (Acharya and Sahoo, 2011; Wu et al., 2011). Their aggregation and rapid clearance have to be avoided (Veiseh et al., 2010), and their pharmacokinetic profile should be studied in advance (Grazú et al., 2012). Equally important, it is extremely difficult for a nanoplatform to selectively reach its target site (Wu et al., 2011). Currently, the most universal way to improve its affinity toward a target is by attachment of ligands [RGD (Zhou et al., 2012), antigen, and folate] that selectively recognize and bind to the target site. This active targeting, which relies on specific interactions, can lead to the accumulation of the nanoplatforms preferentially in, e.g., a tumor region, an ischemic tissue, or an inflamed area as a result of their extravasation through permeable vasculature, an effect known as enhanced permeation and retention (EPR) (Maeda, 2001; Hartner et al., 2009; Albanese et al., 2012).

Multifunctional NPs have been used to design biomaterials and nanoplatforms able to entrap and deliver drugs and biomolecules (such as DNA and growth and differentiation factors, among others bioactive agents) to cells and tissues, for the effective implementation of regenerative therapies (Panyam and Labhasetwar, 2003; Solanki et al., 2008; Grazú et al., 2012). For example, GNPs conjugated with a DNA–polymer complex have been used as nanoscaffolds for delivery of DNA into hMSCs through reverse transfection (Uchimura et al., 2007). They have also been used as a reinforcing- or bioactivity-enhancement phase for polymeric matrices in 3D scaffolds for tissue engineering (Reddy et al., 2006; Engel et al., 2008).

Nanocarriers designed for delivery are able to bypass biological barriers, such as cell membranes and the blood–brain barrier, and can be loaded with high drug concentrations of therapeutics to be released. Once the target site is reached, therapeutic drugs must be delivered from the nanocarrier in order to become bioavailable and aid the regeneration process (Qiu and Park, 2001). Passive processes, such as diffusion, particle erosion, particle degradation, and polymer swelling, can control the release (Mudshinge et al., 2011). The nanoplatform has to be stable enough to promote controlled release of cargo exclusively when triggering (Loomis et al., 2011; Wong and Choi, 2015). Interestingly, the delivery can be activated using *in vivo* signals, such as pH (Bigall et al., 2011; Sato et al., 2011), ion concentration, redox potential (Kang et al., 2010; Luo et al., 2011), presence of certain enzymes (de la Rica et al., 2012), and temperature (Kim and Lee, 2004). Exogenous triggering using nanomaterials responsive to light (Byoung-chan and Kun, 2012), magnetic fields (Ge et al., 2012; Hawkins et al., 2012), or ultrasounds (Epstein-Barash et al., 2010) can be advantageous, since it can control the timing and degree of release (Ganta et al., 2008), thus minimizing the drug release at off-target sites (Rwei et al., 2015).

## Bioimaging Techniques

Bioimaging gives morphological, anatomical, and physiological information of biosamples and reports on the extension of the pathology and organ dysfunction, thus giving valuable information about selecting the best administration route of the elected drug (Naumova et al., 2014; Dong et al., 2015). Different non-invasive imaging techniques, differing in terms of sensitivity, resolution, data acquisition time, penetration depth, and costs, have been explored using NPs (Table 1) (Liu, 2011; Roco et al., 2011; Gao et al., 2013; Naumova et al., 2014; Prodi et al., 2015).

**Table 1:** **Comparison of properties between different imaging techniques**.

Technique	Advantages	Disadvantages	Example of UCNP application
LI	• High sensitivity • High spatial resolution • Short acquisition times • No ionizing radiation • Real-time images • Low cost • Multiplexing	• High scattering • Autofluorescence (NIR light avoids these drawbacks) • Limited penetration depth (cm)	*In vivo* visualization of MCF-7 tumors (Zhu et al., 2014)
RI	• High specificity • High spatial resolution • No photobleaching • No autofluorescence • No scattering • No ionizing radiation	• Limited penetration depth (cm) • Low sensitivity • Long acquisition times	UCF-SERS dual mode tag for living cell and *in vivo* bioimaging (Niu et al., 2014)
CT	• High spatial resolution • Short acquisition times • Unlimited penetration depth • Moderately expensive	• Limited soft tissue discrimination • Health risks (X-Ray)	*In vivo* imaging of different organs in a rat (Liu et al., 2012a)
USI	• High sensitivity • High spatial resolution • Short acquisition times • No ionizing radiation • Real-time images • Low cost	• Limited penetration depth (cm) • Operator dependent	LI and USI dual-modality imaging (Jin et al., 2015)
PAI	• High sensitivity • High specificity • No ionizing radiation • Short acquisition times • Low cost	• Moderate penetration depth	*In vivo* imaging of a mouse (Maji et al., 2014)
MRI	• High specificity • High spatial resolution • No ionizing radiation • High soft tissue contrast • Unlimited penetration depth	• Limited sensitivity • Long acquisition times • Expensive	Angiography and atherosclerotic plaque imaging (Xing et al., 2014)
NI	• High sensitivity • Unlimited penetration depth • Short acquisition times	• Low spatial resolution • Health risks (γ-rays) • Expensive	Lymphatic system images of a mouse (Sun et al., 2011)

### Luminescence Imaging

Light of an external source (UV–VIS–IR light) is absorbed by the contrast (emissive) agent (injected into the cell, tissue, or animal) and subsequently emitted at shorter or longer wavelengths. This low-cost method presents high sensitivity and enables fast analysis. Limitations of luminescence imaging (LI) are high light scattering and autofluorescence of the biological sample when using short wavelengths (UV and VIS light) (Sharma et al., 2006; Prodi et al., 2015; Wolfbeis, 2015).

### Raman Imaging

This is based on the Raman effect, which is the inelastic molecular scattering of incident light (Jokerst et al., 2013). Raman imaging (RI) has high specificity because each chemical bond has a characteristic vibrational energy. The absence of photobleaching and autofluorescence background when using NIR excitation makes this technique very promising in regenerative medicine (Gao et al., 2013). However, its weakness is its low photon efficiency (less than one in a million incident photons corresponds to Raman scattering), resulting in weak signals and/or long acquisition times (Liu, 2011). To increase the signal, the Raman active molecules are placed on a metallic plasmonic surface, usually GNPs, due to their very strong surface enhancement capabilities when illuminated at the plasmon resonance band (Jokerst et al., 2013). This technique is termed surface-enhanced Raman scattering (SERS) (Jokerst et al., 2013).

### X-Ray Computed Tomography

This is one of the most widely used tools in clinical diagnosis due to its availability, efficiency, and low cost. It directs X-rays to a biosample and measures the decrease in intensity along a linear path, obtaining cross sectional images (Law and Wong, 2014). Computed tomography (CT) can provide anatomic and functional information of bones, organs, and tissues and is generally used in combination with other techniques, such as MRI and PET (Law and Wong, 2014). CT requires short acquisition time, has an unlimited penetration depth, and high spatial resolution; however, its weakness is that it has a limited soft tissue discrimination, and there are concerns on the health risks associated with X-ray radiation (McMahon and Currell, 2013).

### Ultrasound Imaging

These images are generated from pulsed sound waves reflected and transmitted between tissue structures, which are eventually detected as echoes (time response to travel back) (Naumova et al., 2014). This technique allows real-time images, presents high sensitivity, and is low cost. However, its disadvantage is its limited penetration depth in tissues. In spite of that, this is one of the most commonly used techniques in clinical assays (Law and Wong, 2014).

### Photoacoustic Imaging

The absorbed energy from an external source (VIS–IR light) is transformed into kinetic energy of the sample through energy exchange processes, i.e., the incident light is converted into ultrasonic emission (Gao and Li, 2016). This technique hybridizes the high contrast and spectral selectivity of optical imaging with high ultrasonic resolution. It is a low cost and rapid technique, but has a moderate penetration depth (Prodi et al., 2015).

### Magnetic Resonance Imaging

It is based on the principles of nuclear magnetic resonance, which uses magnets to polarize the hydrogen nuclei in water molecules, thus obtaining a spatial distribution of signals emitted from protons in the tissue (Law and Wong, 2014). MRI is a popular technique for cellular imaging in large animals and humans due to its high temporal and spatial contrast, high specificity, absence of ionizing radiation, and unlimited penetration depth. Unfortunately, MRI is expensive and needs a long time of analysis (Yeo et al., 2014).

### Nuclear Imaging

Positron emission tomography and single photon emission computed tomography (SPECT) use radionuclides (Law and Wong, 2014). PET uses biologically active positron-emitting radiotracers (such as fluorine-18) that decay and cause the annihilation of a positron and an electron producing two γ-rays. This is one of the most sensitive methods for quantitative measurement of physiologic processes *in vivo* (Law and Wong, 2014). SPECT consists of the emission of positrons to emit a single γ-ray, which is measured directly by rotating gamma cameras obtaining the image (Law and Wong, 2014). These methodologies possess high sensitivity and no limitations in penetration depth. The main drawbacks are the problems caused by the radiation, long acquisition times, and the presence of artifacts easily generated due to patient movement or bad distribution of the radiotracer (Law and Wong, 2014; Naumova et al., 2014).

## Upconversion Nanoparticles in Bioimaging

Upconversion nanoparticles upconvert NIR light (800, 915, and 980 nm) to multi-wavelength light (narrow emission bands at UV, visible, and even NIR region) using a low-power continuous-wave diode laser. NIR excitation allows for deep tissue penetration and avoids autofluorescence of the biological samples (i.e., higher sensitivity) (Philippot and Reiss, 2012; Dong et al., 2015). Moreover, the possibility of using both NIR excitation and NIR emission (NIR-to-NIR upconversion) is particularly relevant for *in vivo* imaging of small animals, because it permits deep tissue penetration and less absorption and scattering of bio-tissues and organs. These properties combined with their high stability, low cytotoxicity (Gnach et al., 2015; Sun et al., 2015), good photostability, and non-photoblinking or -photobleaching make UCNPs unique optical tools for biological studies (Liu, 2011; DaCosta et al., 2014; Bünzli, 2015; Dong et al., 2015; Prodi et al., 2015). The only drawback of UCNPs in LI is their relatively low upconversion quantum yield; several strategies are being explored to enhance their emission yield (such as covering the UCNPs with an inorganic shell) (Chen et al., 2014a, 2015).

Though UCNPs have been mainly used in LI, recent studies have shown their versatility for other bioimaging techniques. PEG-capped NaYbF_4_:Er^3+^, Gd^3+^ NPs have been effectively internalized by HeLa cells and provided higher contrast images of a rat heart than equivalent concentrations of iobitridol (a current clinical contrast agent) (Liu et al., 2012a). Moreover, UCNPs can be conveniently functionalized with iodinated compounds (silica-coated UCNPs with an iodine compound attached that absorbs X-ray radiation) to make them suitable for CT imaging (Zhang et al., 2011). These UCNPs allowed the visualization of a liver for 30 min, thus demonstrating their long circulating time. Moreover, hybrid BaYbF_5_ NPs perform well in the *in vivo* X-ray CT angiography (Liu et al., 2012b). This binary CT agent was more efficient than iobitridol and allowed the visualization of the vasculature in an *in vivo* mouse model during 2 h.

Upconversion nanoparticles have also been studied as potential photoacoustic imaging (PAI) agents (Maji et al., 2014), using NaYF_4_:Yb^3+^, Er^3+^ NPs covered with α-cyclodextrin. Excitation at 980 nm of the nanosystem dispersed in water led to luminescence quenching due to non-radiative relaxation processes and brought about an enhanced photoacoustic signal. The *in vivo* localization of the UCNPs in mice showed their capability for PAI (Maji et al., 2014).

In addition, UCNPs containing Gd^3+^ as dopant and/or as host matrix have been studied as MRI contrast agents. For example, ultra-small NaGdF_4_ NPs have proved more efficient in MRI angiography and atherosclerotic plaque imaging than some commercial MRI contrast agents, and they were easily excreted by kidney (Xing et al., 2014). Furthermore, a chelating molecule [diethylenetriaminepentaacetic acid (DTPA)] was used to functionalize the surface of UCNPs with the aim of capturing potentially released Gd^3+^ ions, thus avoiding toxic effects *in vivo* (Xing et al., 2014). Also, Gd^3+^ can be incorporated in both, the NP as dopant ion and the ligand. Recent studies demonstrated that simultaneous internal and external incorporation of Gd^3+^ ions increase MRI sensitivity (Du et al., 2016).

Moreover, UCNPs can be used for multimodal imaging, i.e., the combination of two or more imaging techniques to benefit from their strengths and weaknesses (Chen et al., 2014a; DaCosta et al., 2014; Li et al., 2014a; Bünzli, 2015; Cheng and Lin, 2015; Christ and Schäferling, 2015; Dong et al., 2015; Park et al., 2015; Prodi et al., 2015; Rieffel et al., 2015a; Wu et al., 2015; Zhou et al., 2015). There are examples of UCNPs in bimodal imaging combining PET/LI (Sun et al., 2011), SPECT/LI (Yang et al., 2013), PAI/LI (Maji et al., 2014), MRI/LI (Zhou et al., 2010; Cheng et al., 2013a), CT/LI (Zhang et al., 2011; Liu et al., 2013a; Zheng et al., 2014), and ultrasound imaging (USI)/LI (Jin et al., 2015).

Thus, NaGdF4:Yb^3+^, Er^3+^, NPs functionalized with bovine serum albumin attached to DTPA-Gd^3+^ have proved useful in upconversion LI and MRI (Du et al., 2016). ^18^F radionuclide has been directly bound to the surface of NaYF_4_ UCNPs to generate a dual-model bioimaging technique, combining upconversion LI and PET imaging (Sun et al., 2011). Several prevalent diseases are associated with the lymphatic system, which is usually difficult to study by bioimaging due to the lack of techniques with adequate sensitivity and temporal resolution. Recently, NaYF_4_:Yb, Tm UCNPs have been labeled with ^18^F^−^, and the *in vivo* mouse distribution of ^18^F-NaYF_4_:Yb, Tm UCNPs was monitored by upconversion LI. In addition, the mouse lymphatic system was imaged with ultra-high sensitivity by PET (Sun et al., 2011). Moreover, a nanohybrid comprising NaYF_4_:Yb, Er, and Fe_3_O_4_ NPs has shown versatile for upconversion LI and MRI. Mouse MSCs (mMSCs) were labeled with this nanohybrid, and the resulting nanoplatform was injected in mice with two wounds at opposite sides on the abdominal skin. Then, a magnet was placed on one of the wounds for 6 h to target magnetically the mMSCs at one of the wounds. The mMSCs were followed *in vivo* by LI and MRI, thus observing that the wound treated with mMSCs presented enhanced tissue repairing (Cheng et al., 2013a).

In addition, examples of trimodal (Xing et al., 2012; Cheng et al., 2013b; Ni et al., 2014; Wang et al., 2014a; Yi et al., 2015; Zhai et al., 2015), tetramodal (Sun et al., 2013), and even hexamodal (Rieffel et al., 2015b) imaging by using UCNPs have also been reported.

## Upconversion Nanoparticles in Regenerative Medicine

**Figure 1:**
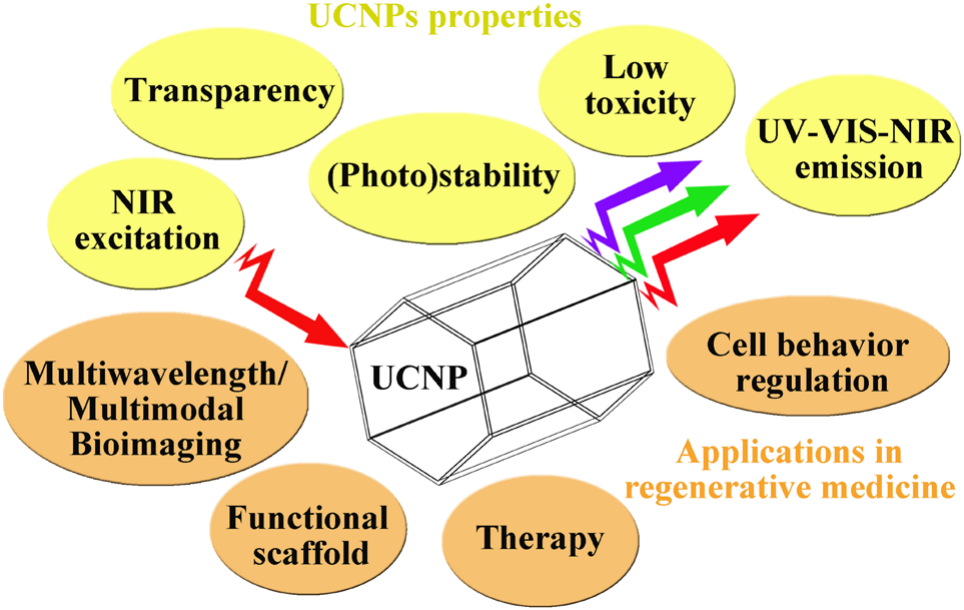
**Overview of UCNPs properties and reasons to apply them in regenerative medicine**.

Near-infrared light is particularly attractive to monitor cell–surface interactions in regenerative medicine (Figure 1). NIR-controlled cell adhesion has been achieved with UCNP-based programmable substrates either by using photocaged linkers (Li et al., 2014b) or *via* photoswitchable substrates (Li et al., 2015). The UCNP harvested NIR light and converted it into UV light, thus inducing the cleavage of the photocaged linkers and on-demand release of adhesive cells (Li et al., 2014b). This strategy not only enables deep tissue photocontrol of the cell adhesion on substrate but also opens a new approach to design UCNP-based cell scaffolds to manipulate dynamically cell–matrix and cell–cell interactions (Li et al., 2014b).

**Figure 2:**
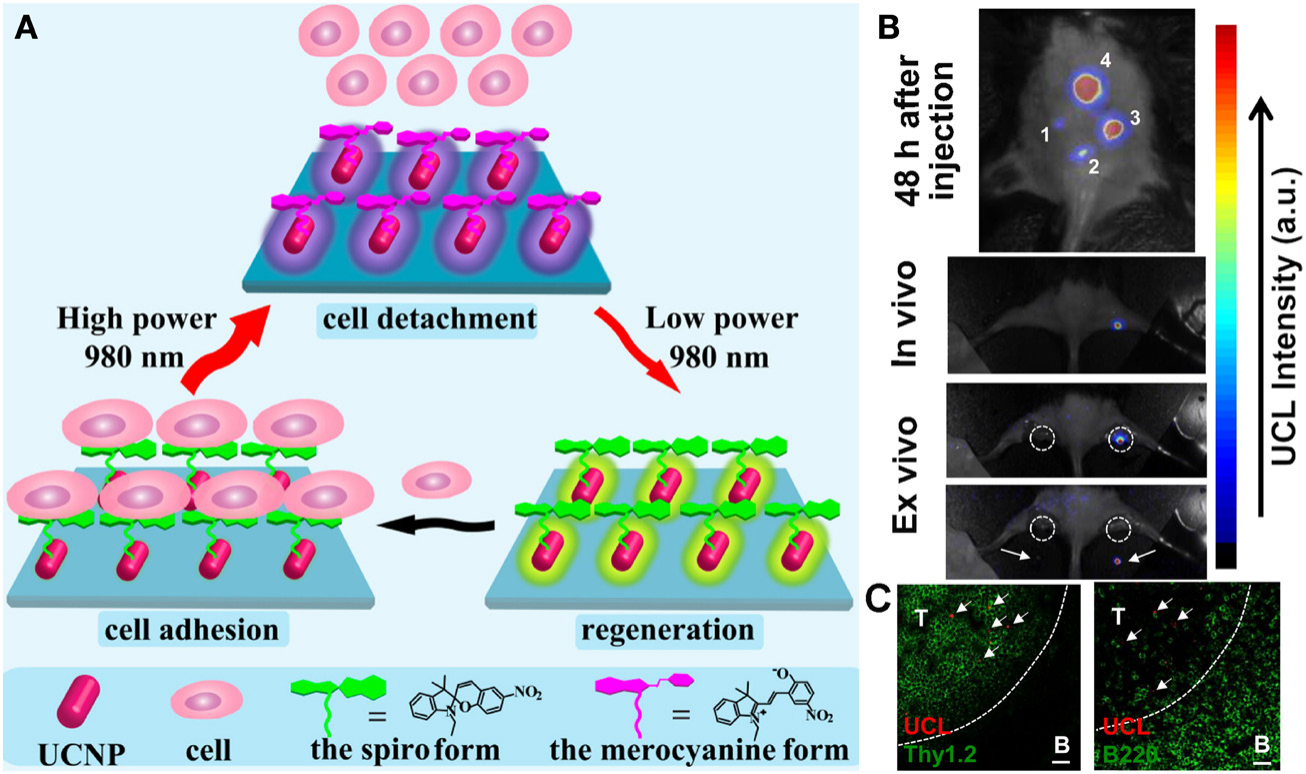
**(A)** Schematic illustration of the utilization of SP-UCNP as a NIR-triggered photoswitch for non-invasive and reversible control of cell adhesion/detachment by merely altering the power density of a single-wavelength 980-nm laser. Reprinted with permission from Li et al. (2015). Copyright (2015) American Chemical Society (Li et al., 2015). **(B)** *Top*: UCL image of a C56BL/6 mouse subcutaneously injected with various numbers of UCNPs labeled DCs (≈50–50,000). *Middle*: labeled DCs were injected into the rig; strong UCL signals from the draining lymph node were seen under *in vivo* UCL imaging. *Bottom*: *ex vivo* imaging of popliteal lymph nodes (white circles) before (top) and after (bottom) being dissected from the mouse. **(C)** Immunofluorescence images of the lymph nodes dissected from the mouse injected with UPP@OVA-labeled DCs. T: T-cell zone (Thy1.2^+^). B: B-cell zone (B220^+^). Scale bar = 20 μm. Arrows point to UCL signals from labeled DCs. Adapted with permission from Xiang et al. (2015). Copyright (2015) American Chemical Society (Xiang et al., 2015).

Cell-molecular recognition can be reversibly guided in interfacial systems by using photoswitching systems based on photoisomerization reactions (Weber et al., 2014). In this context, UCNPs have been combined with photoswitchable systems such as spiropyrans (which converts to merocyanine after ring opening) (Zhang et al., 2012a,b; Chen et al., 2014b; Lai et al., 2014; Zhou et al., 2014), as well as diarylethenes (Boyer et al., 2012; Yang et al., 2014) and azobenzenes (*cis*/*trans*-isomerization) (Liu et al., 2013b; Wang et al., 2014b). Recently, nanohybrids consisting of core–shell–shell–shell UCNPs, specifically, NaYF_4_:Tm,Yb@NaYF_4_@NaYF_4_:Er,Yb@NaYF_4_ NPs were coated with silica and conjugated afterward with spiropyran (Figure 2A). This system has been described as a new generation of single-wavelength NIR-controlled photoswitches, which can control the efficient adhesion and detachment of cells reversibly and non-invasively (Li et al., 2015).

Upconversion nanoparticles are unique for live cell tracking because of their optical properties (Vetrone et al., 2010; Tian et al., 2012; Li et al., 2013). Thus, silica/NaYF_4_:Yb, Er NPs loaded into cells were applied in cell migratory tracking for 5 h by a time-lapse confocal microscope. The direction, speed, and cell–cell interaction of migrating cells were clearly visualized. Consequently, the nanoplatform could be used to track live myoblast cells in a living mouse model with cryoinjured hind limb (Idris et al., 2009). Indeed polymer-coated UCNPs with different emission colors, aimed to multicolor *in vivo* upconversion LI, well performed in multiplexed lymph node mapping, and multicolor *in vivo* cancer cell tracking (Cheng et al., 2010).

Even though UCNPs have relatively low upconversion efficiencies, they have shown ultrahigh sensitivity *in vivo* stem cell tracking (Solanki et al., 2008; Cheng et al., 2013a; Gao et al., 2013). In addition, antigen-loaded UCNPs have recently been used to label and stimulate dendritic cells (DCs) to induce antigen-specific immune response *in vivo* animals (Figures 2B,C). The homing of DCs to draining lymph nodes was monitored by impressively sensitive *in vivo* tracking of the NP-labeled DCs by upconversion LI (Xiang et al., 2015); a few DCs were enough to be clearly seen, while, comparatively, thousands of cells are usually needed to enable *in vivo* tracking in mice when using QDs and MNPs (Kraitchman et al., 2005; Yukawa et al., 2010).

It is worth noting that UCNPs can be used as multifunctional nanoplatforms for light-driven therapies, such as photothermal and photodynamic therapy, and spatiocontrolled drug delivery (Chen et al., 2014a; Shanmugam et al., 2014; Rwei et al., 2015). Therefore, it is presumed that they will become relevant in cell therapy, cell therapy combined with bioimaging, and by extension in regenerative medicine.

## Conclusion

Upconversion NPs have unique properties for their application in regenerative medicine. Specifically, they are considered non-toxic; can exhibit NIR-to-visible upconversion luminescence and, more importantly, NIR-to-NIR upconversion luminescence; possess extraordinary (photo)stability; and can be designed with additional features to make them useful tools for other bioimaging techniques or for multimodal imaging. Moreover, UCNPs are excellent candidates for multifunctional NP therapeutics, which can be triggered locally or remotely by NIR light. Thus, UCNPs can be relatively easily visualized in the body and can monitor the biological components and events, and/or may behave as functional scaffolds in the regeneration of damaged tissues and/or organs. Undoubtedly, UCNPs can be used for selective treatment of the affected area using photonics with simultaneous visualization of the process due to their multi-wavelength emission.

## Author Contributions

All authors listed, have made substantial, direct and intellectual contribution to the work, and approved it for publication.

## Conflict of Interest Statement

The authors declare that the research was conducted in the absence of any commercial or financial relationships that could be construed as a potential conflict of interest.
